# The A_2B_ adenosine receptor in MDA-MB-231 breast cancer cells diminishes ERK1/2 phosphorylation by activation of MAPK-phosphatase-1

**DOI:** 10.1371/journal.pone.0202914

**Published:** 2018-08-29

**Authors:** Marthe Koussémou, Kristina Lorenz, Karl-Norbert Klotz

**Affiliations:** 1 Institut für Pharmakologie und Toxikologie, Universität Würzburg, Würzburg, Germany; 2 Leibniz-Institut für Analytische Wissenschaften–ISAS–e.V., Bunsen-Dortmund, Germany, and West German Heart and Vascular Center Essen, Essen, Germany; University of South Alabama Mitchell Cancer Institute, UNITED STATES

## Abstract

It was previously shown that the estrogen-receptor negative breast cancer cell line MBA-MD-231 expresses high levels of A_2B_ adenosine receptors as the sole adenosine receptor subtype. These receptors couple to both, stimulation of adenylyl cyclase and a Ca^2+^ signal. In order to establish a potential role of A_2B_ adenosine receptors in tumor growth and development MAPK signaling was investigated in these breast cancer cells. Although it is known that A_2B_ adenosine receptors may stimulate MAPK it was found that in MBA-MD-231 cells ERK1/2 phosphorylation is reduced upon agonist-stimulation of A_2B_ adenosine receptors. This reduction is also triggered by forskolin, but abolished by the PKA inhibitor H89, suggesting an important role for the cAMP-PKA pathway. Likewise, a role for intracellular Ca^2+^ was established as the Ca^2+^ chelator 1,2-bis-(o-aminophenoxy)-ethane-*N*,*N*,*N*’,*N*’-tetraacetic acid, tetraacetoxymethyl ester (BAPTA-AM) abolished the reduction of ERK1/2 phosphorylation triggered by A_2B_ stimulation. It was shown that various pathways downstream from A_2B_ adenosine receptors resulted in a stimulation of MAPK phosphatase-1 (MKP-1) which dephosphorylates phospho ERK1/2, and thus plays a critical role in the regulation of the phosphorylation state of ERK1/2. The reduction of ERK1/2 phosphorylation mediated by A_2B_ adenosine receptors might provide an interesting approach for adjuvant treatment leading to reduced growth of certain tumors expressing the A_2B_ subtype.

## Introduction

Adenosine receptors (ARs) comprise a family of four G protein-coupled receptors which are identified as A_1_, A_2A_, A_2B_, and A_3_ subtypes [1, 2]. The canonical signal of the A_1_ and A_3_ subtypes is inhibition of adenylyl cyclase whereas the A_2_ subtypes mediate a stimulation of cAMP production. Adenosine affects the function of virtually every organ due to abundant expression of one or several AR subtypes [1–3]. Consequently, adenosine receptors are considered as interesting drug targets for the treatment of numerous conditions including cardiovascular, inflammatory and neurological diseases [3, 4]. Increasing evidence suggests that adenosine may also be involved in the regulation of cellular growth control and proliferation via all four receptor subtypes and, therefore, these may serve as therapeutic targets to combat tumor development and growth [5–8].

We have previously characterized the expression of adenosine receptors in the estrogen receptor-negative breast cancer cell line MDA-MB-231 [9]. These cells express A_2B_ adenosine receptors as the sole subtype at a very high density of > 1 pmol/mg membrane protein [9]. In an attempt to characterize a potential pathophysiological role of this highly expressed AR subtype we characterized MAPK signaling in these cells and discovered that stimulation of A_2B_ARs causes a reduction of ERK1/2 phosphorylation. Stimulation of A_2B_ARs might therefore help to control growth and proliferation of these and other cancer cells expressing A_2B_ARs. The goal of this study was to understand the signaling pathway(s) leading to the inhibition of MAPK signaling as shown by a reduction of ERK1/2 phosphorylation as a consequence of stimulation of A_2B_ARs.

The observed reduction of ERK1/2 phosphorylation might be the result of inhibition of kinases upstream from ERK1/2. One well-known GPCR-G_s_-meditated signalling pathway that can lead to ERK1/2 inhibition and anti-proliferative effects in cancer cells is for example the cAMP-PKA-mediated Raf1 inhibition via PKA-phosphorylation at serine 259 [10–12]. Alternatively, the stimulation of a phosphatase could be responsible for the inhibitory input on MAPK signaling in MDA-MB-231 breast cancer cells [13, 14]. We found that several A_2B_AR mediated signaling pathways control MKP-1 activity which plays an important role in the regulation of ERK1/2 phosphorylation and, thus, may control growth and proliferation of these cells.

## Materials and methods

### Materials

The breast cancer cell line MDA-MB-231 was provided by the Institut für Zellbiologie (Tumorforschung), Universitätsklinik Essen, Germany. Cell culture media and fetal calf serum were purchased from PanSystems, Aidenbach, Germany. Penicillin (100 U/ml), streptomycin (100 μg/ml), L-glutamine and G-418 were from Gibco-Life Technologies, Eggenstein, Germany. *N*-[2-[[3-(4-Bromophenyl)-2-propenyl]amino]ethyl]-5-isoquinolinesulfonamide (H-89) and BAPTA-AM were purchased from Calbiochem, Bad Soden, Germany. All inhibitors were used at concentrations at least 10 fold higher than reported IC_50_ values. The adenosine receptor ligands 9-ethyl-8-furyl adenine (EFA, antagonist) and HEMADO (agonist) were provided by Prof. Rosaria Volpini, School of Pharmacy, Medicinal Chemistry Unit, University of Camerino. Other adenosine receptor agonists and antagonists were from Sigma/RBI, Taufkirchen, Germany and cAMP derivatives from BioLog Life Science Institute, Bremen, Germany. Sources for antibodies were as follows: anti-phospho-ERK1/2 and anti-ERK1/2, New England Biolabs, Frankfurt, Germany; anti MKP-1 and anti-GAPDH, Santa Cruz, Heidelberg, Germany; anti-β-tubulin, Sigma, Taufkirchen, Germany; secondary HRP-conjugated goat anti-rabbit antibodies, Dianova, Hamburg, Germany. Other materials were obtained from sources as described earlier [9, 15].

### Cell culture

MDA-MB-231 breast cancer cells were grown adherently and maintained at 37°C in DMEM containing 10% fetal calf serum, penicillin (100 U/ml), streptomycin (100 μg/ml), L-glutamine (2 mM) in 5% CO_2_/95% air. Cells were split 2 or 3 times weekly at a ratio between 1:4 to 1:8 [9]. Fetal calf serum was omitted on the day of the experiment.

### Immunoblot analysis

Prior to an experiment, confluent MDA-MB-231 cells were washed with PBS, then trypsinized and seeded at 50,000 cells/well in precoated six well plates in medium containing 10% fetal calf serum for 24 h and then treated with the indicated inhibitors for the respective time periods. When inhibitors were used, they were added 30 min prior to an agonist. Agonist was added and incubated for 30 min. After washing them with ice-cold PBS cells were lysed in electrophoresis sample buffer for 45 min on a shaker at room temperature followed by sonification. Proteins were separated by SDS polyacrylamide gel electrophoresis and transferred to nitrocellulose membranes. For the immunoblot analysis, the membranes were blocked with 5% dry milk powder on a shaker at room temperature and incubated overnight at 4°C with the appropriate primary antibody. Equal loading was confirmed with GAPDH (see S1 Fig) or β-tubulin antibody. After incubation with horseradish peroxidase–coupled secondary antibody, antigen-antibody complexes were visualized by the ECL method and quantified by densitometry with ImageJ (for details see [16]).

### Data analysis

All experiments were repeated 4–10 times. Statistical difference between two groups of data was tested by the paired Student’s t test and the difference among three or more groups was assessed by a one-way Anova followed by a Bonferroni post-test. These analyses were performed with the statistical software package GraphPad Prism 6.0 (GraphPad Software Inc., La Jolla, CA, USA) and a 0.05 significance level was assumed to indicate statistical significance. Results are expressed as treated/untreated ratio (mean ± S.E.M).

## Results

**Fig 1:**
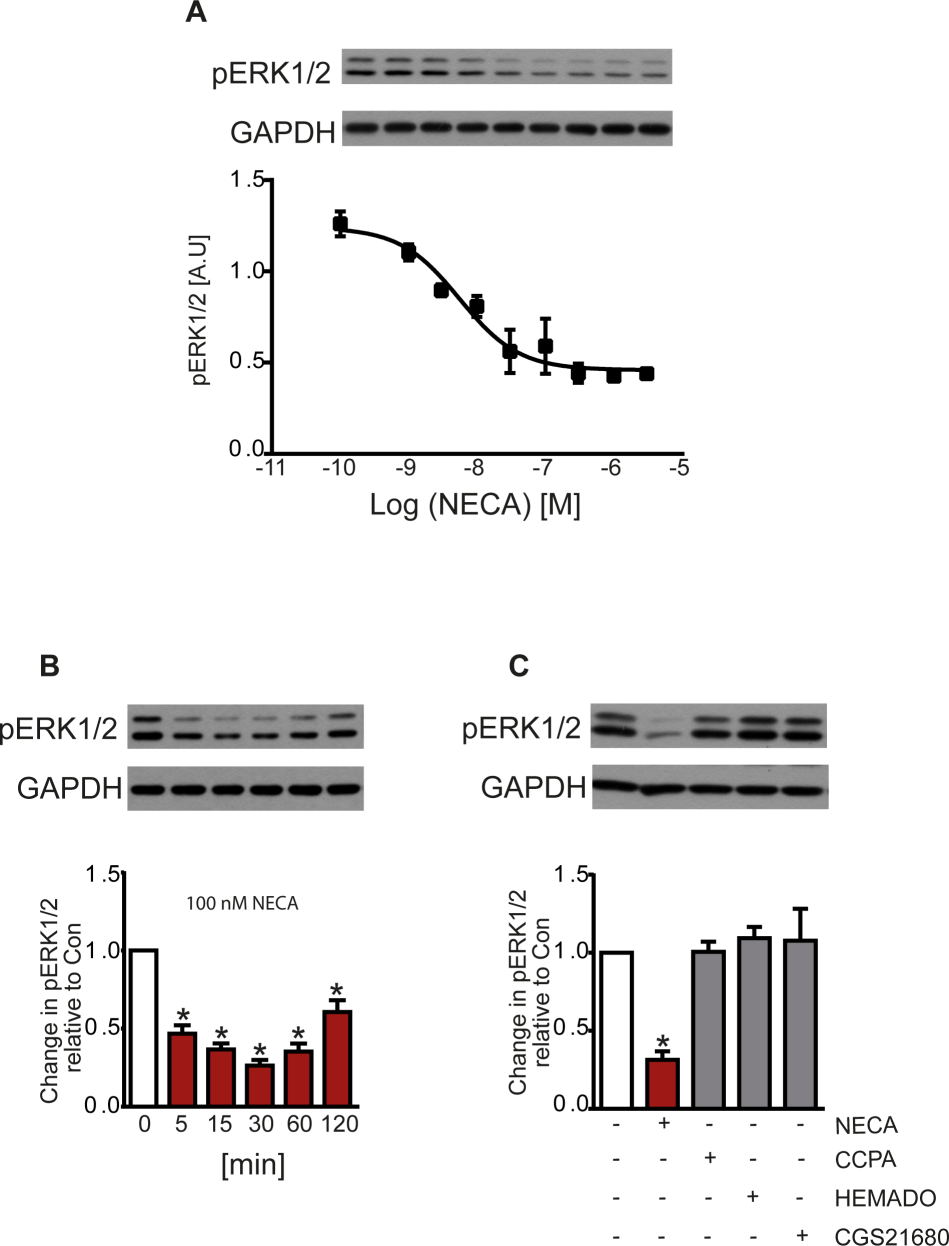
Reduction of ERK1/2 phosphorylation by the adenosine receptor agonist NECA. (A) NECA reduced the ERK1/2 phosphorylation in a concentration-dependent manner (EC_50_ 5.85 nM, 95% confidence limit 2.91–11.8). (B) The reduction was time-dependent with a maximum after 30 min. (C) Only NECA as a nonselective agonist showed this effect while activation of A_1_ with CCPA, A_2A_ (also A_1_ and A_3_ to a certain degree) with CGS 21680, and A_3_ with HEMADO showed no effect. Panel A and the Western blots in B and C show representative experiments, the columns in B and C show data from n = 7 and 5 independent experiments, respectively (* p < 0.001, significantly different from control).

We have previously shown that MDA-MB 231 breast cancer cells express very high levels of A_2B_ receptors as the sole adenosine receptor subtype [9]. In addition to the canonical stimulation of adenylyl cyclase an A_2B_-mediated Ca^2+^ signal was observed in these cells. In this study, we investigated a potential activation of MAP kinase signaling via A_2B_ receptors as a signaling pathway known to be activated by this receptor subtype [17]. As shown in Fig 1, MDA-MB 231 cells exhibited a considerable basal ERK1/2 phosphorylation which was reduced by the nonselective adenosine receptor agonist adenosine-5’-*N*-ethyluronamide (NECA) in a concentration and time dependent manner (Fig 1A and 1B). In contrast, no such reduction was observed with the subtype selective agonists CCPA (A_1_) and HEMADO (A_3_) (Fig 1C). The AR agonist 2-[p-(2-carboxyethyl)phenylethylamino]adenosine-5’-*N*-ethyluronamide (CGS 21680) that does not stimulate the A_2B_ subtype, did also not show any effect (Fig 1C). These data support the notion that the observed reduction of ERK1/2 phosphorylation was mediated by the A_2B_ adenosine receptor.

**Fig 2:**
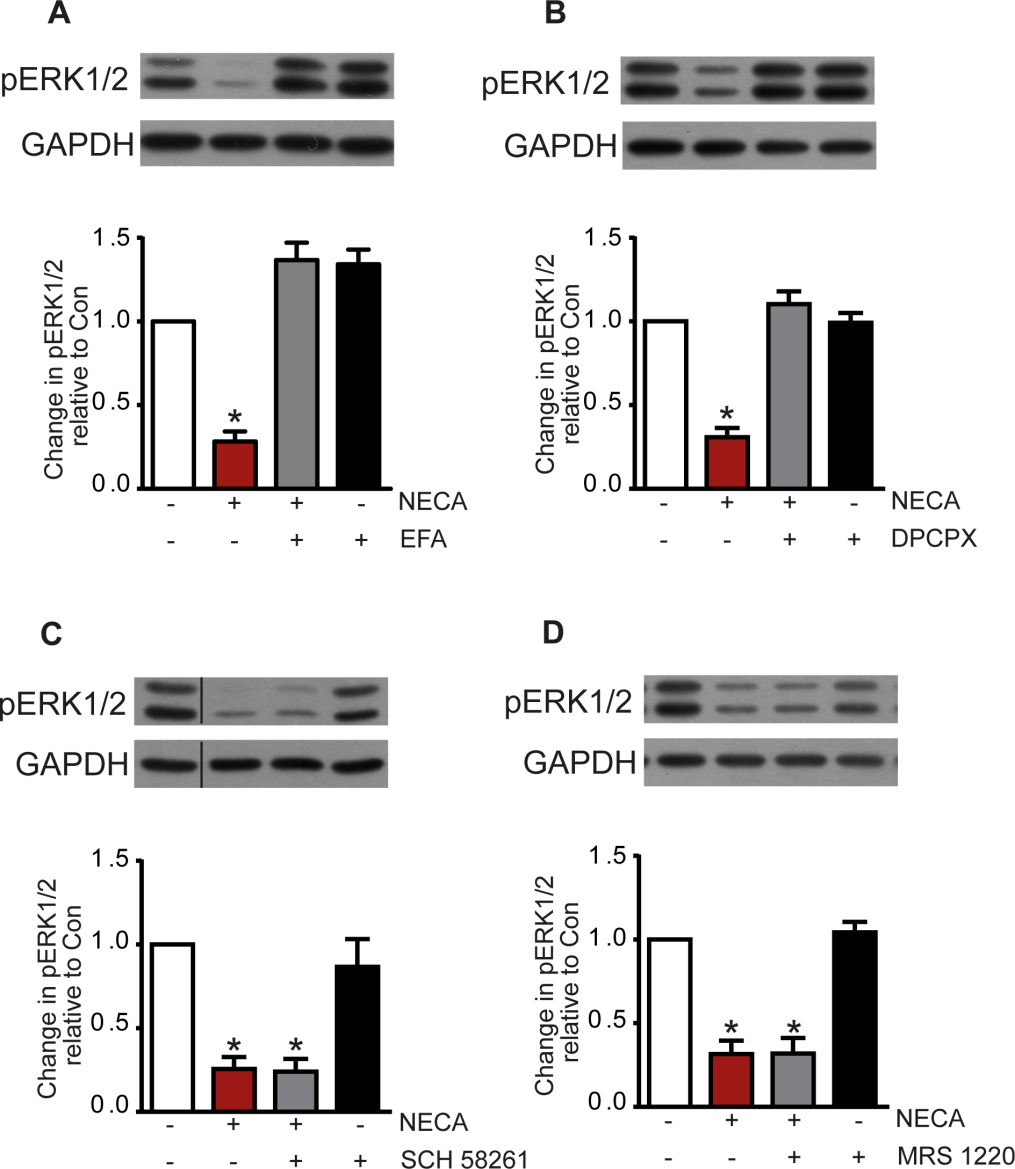
NECA-induced reduction of ERK1/2 phosphorylation and effect of adenosine receptor antagonists. The nonselective antagonist EFA (10 μM) blocked the NECA (100 nM) effect (A) and so did DPCPX at 10 μM, a concentration high enough to block A_2B_AR (B). In contrast, the A_2A_ selective antagonist SCH 58261 (C) and the A3 selective antagonist MRS 1220 (D), both at 10 μM, had no effect. Western blots show a representative experiment, the columns represent mean values of n = 8 (A), 6 (B), 4 (C), and 5 (D) independent experiments (* p < 0.001, significantly different from control).

This suggestion was further confirmed by the pharmacological profile of adenosine receptor antagonists. The NECA-induced reduction of ERK1/2 phosphorylation was antagonized by the nonselective adenosine receptor antagonist EFA (Fig 2A) and the A_1_/A_2B_ antagonist 8-cyclopentyl-1,3-dipropylxanthine (DPCPX) (Fig 2B) with an IC_50_ indicative of an A_2B_ receptor-mediated effect. As shown in Fig 2C and 2D, both the A_2A_ selective antagonist 2-(2-furanyl)-7-(2-phenylethyl)-7*H*-pyrazolo[4,3-*e*][1,2,4]triazolo[1,5-*c*]pyrimidin-5-amine (SCH 58261) and the A_3_ selective antagonist *N*-[9-chloro-2-(2-furanyl)[1,2,4]-triazolo[1,5-*c*]quinazolin-5-yl]benzene acetamide (MRS 1220) had no effect corroborating the A_2B_-mediated effect on ERK1/2 phosphorylation.

**Fig 3:**
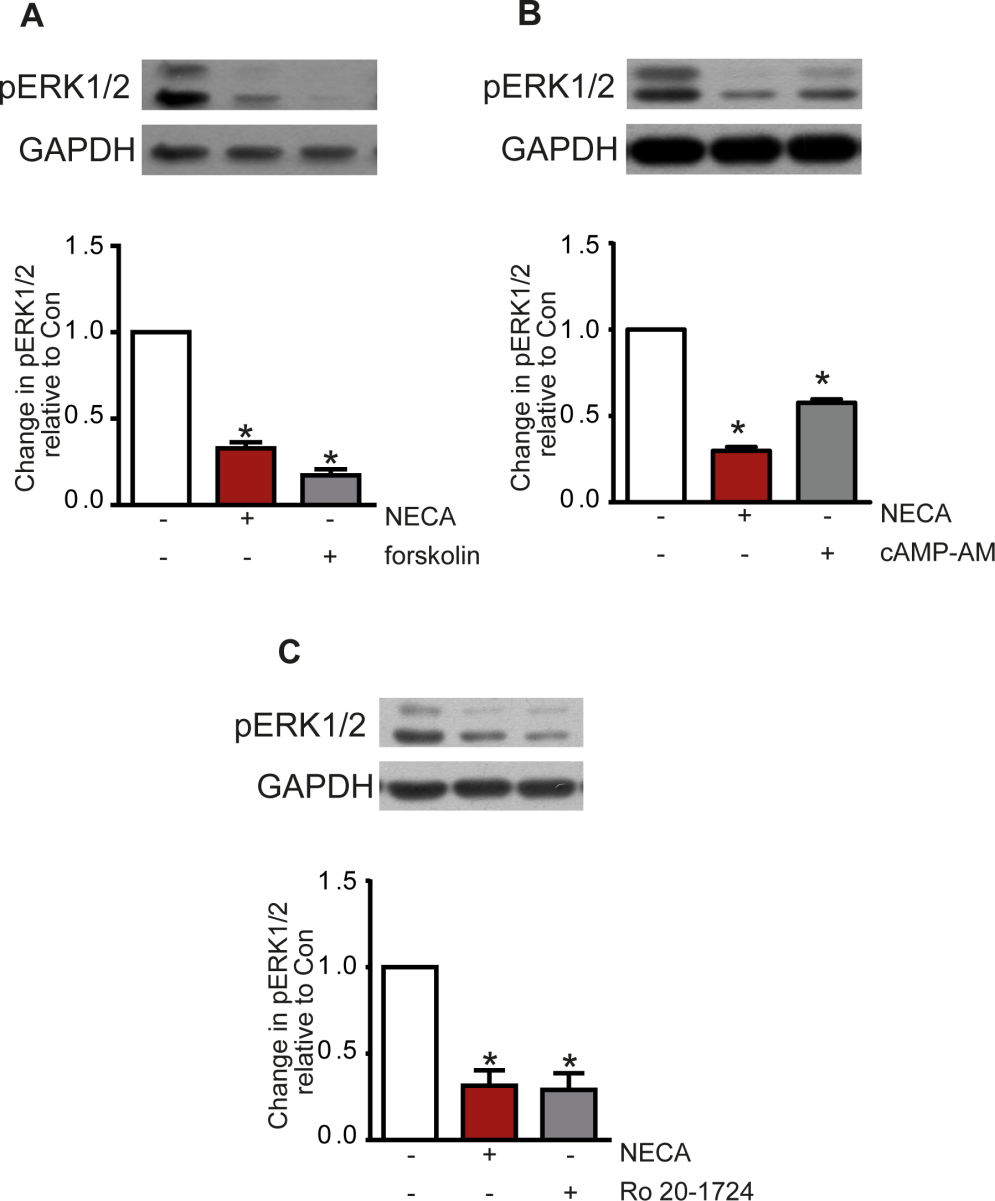
Reduction of ERK1/2 phosphorylation by cAMP. The effect of 100 nM NECA on ERK1/2 phosphorylation was mimicked by compounds increasing intracellular cAMP, like 1 μM forskolin (A), 100 μM cAMP-AM (“caged cAMP”, B), or the PDE inhibitor Ro20-1724 (C) at 100 μM. Western blots show a representative experiment, the columns represent mean values from n = 6 (A), 4 (B), and 5 (C) independent experiments (* p < 0.001, significantly different from control).

**Fig 4:**
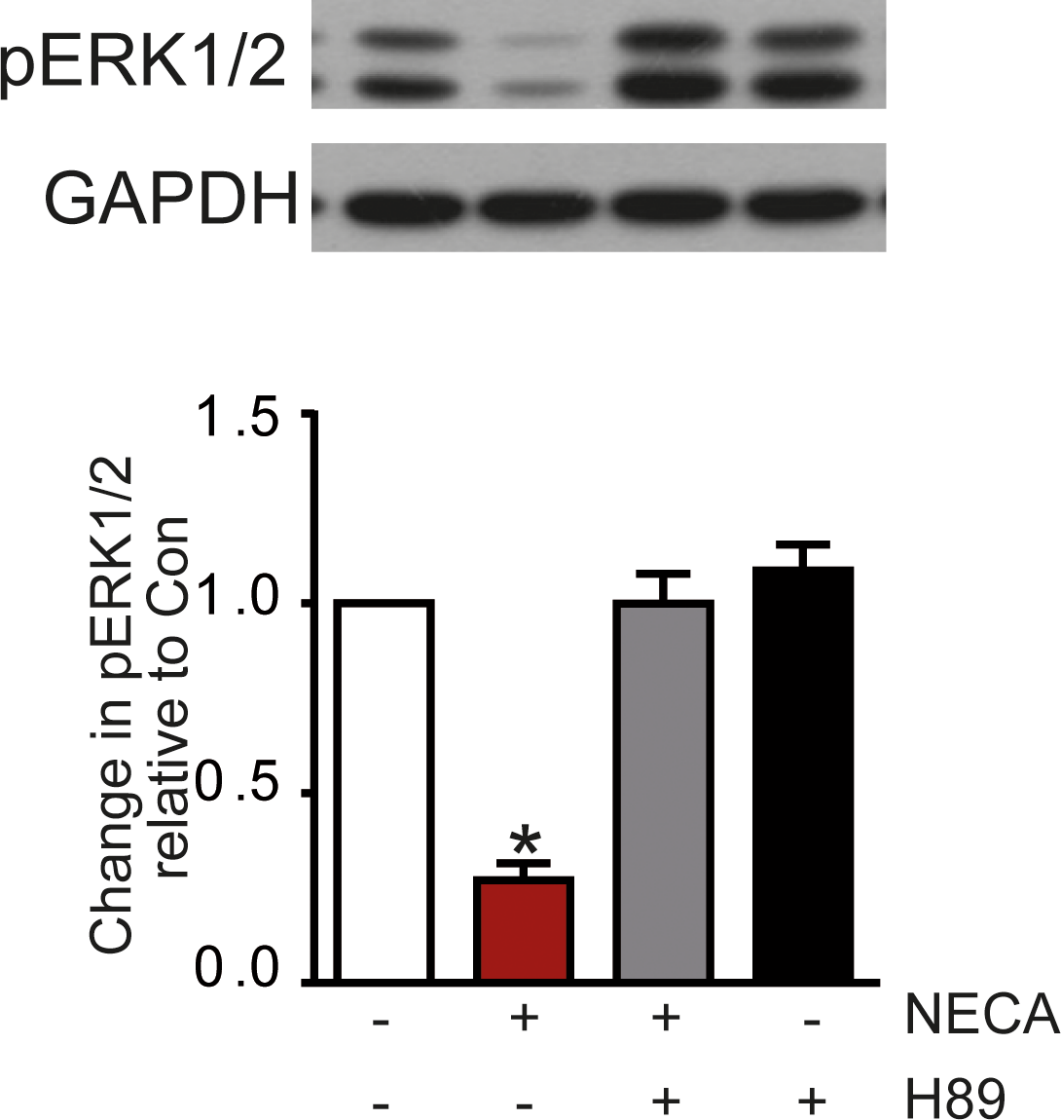
Effect of the PKA inhibitor H-89 on NECA-mediated reduction of ERK1/2 phosphorylation. Preincubation (30 min) of MDA-MB-231 cells with 10 μM of the PKA inhibitors H-89 prevented the NECA effect on ERK1/2 phosphorylation. The Western blot shows a representative experiment, the columns represent mean values from n = 7 independent experiments (* p < 0.001, significantly different from control).

Fig 3A shows that reduction of ERK1/2 phosphorylation was also achieved with forskolin suggesting the involvement of cAMP in the effect. This was further confirmed using the membrane-permeable cAMP analogue adenosine-3’,5’-cyclic monophosphate, acetoxymethyl ester (cAMP-AM, ‘caged cAMP’) which releases cAMP after activation by esterases inside the cell (Fig 3B). Also, inhibition of phosphodiesterase-4 with 4-(3-butoxy-4-methoxyphenyl)methyl-2-imidazolidone (Ro 20–1724) resulted in a reduction of ERK1/2 phosphorylation comparable to the NECA-effect (Fig 3C) further supporting the assumption that cAMP is mediating the effect. The subsequent activation of PKA seems to be necessary as inhibition of PKA with H89 blocked the NECA-induced effect on ERK1/2 phosphorylation (Fig 4).

**Fig 5:**
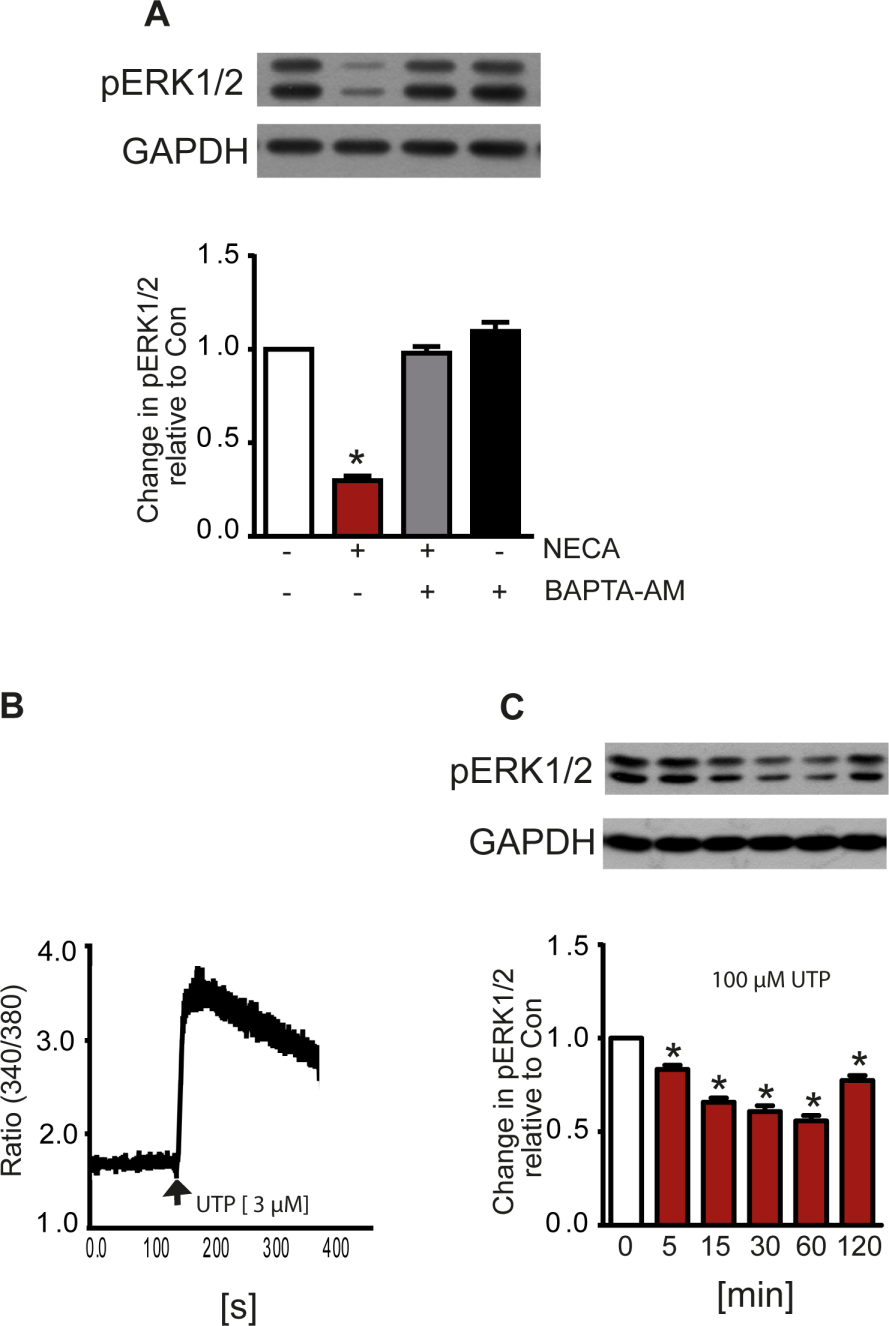
Role of Ca^2+^ for reduction of ERK1/2 phosphorylation. The NECA-induced reduction of ERK1/2 phosphorylation was blocked by the Ca^2+^-chelator BAPTA intracellularly released from 30 μM of the cell-penetrating derivative BAPTA-AM (A). Consequently, an intracellular increase of Ca^2+^ triggered by 100 μM UTP (B) resulted in a reduction of ERK1/2 phosphorylation similar to increasing levels of cAMP (C). Western blots and the Ca^2+^ trace in B show a representative experiment, the columns represent mean values from n = 7 (A) and 8 (C) independent experiments (* p < 0.001, significantly different from control).

We have previously shown that stimulation of the A_2B_ receptor in MDA-MB-231 cells triggers a Ca^2+^-response in addition to the activation of adenylyl cyclase (Panjehpour et al., 2005). Therefore, we investigated whether Ca^2+^ signaling might be involved in the NECA-induced reduction of ERK1/2 phosphorylation. As shown in Fig 5A the chelator BAPTA (applied as the cell-permeable precursor BAPTA-AM) completely abolished the NECA effect. Consequently, triggering a Ca^2+^-signal with the P2Y_2/4_ agonist UTP (Fig 5B) resulted in a time-dependent reduction of ERK1/2 phosphorylation (Fig 5C).

**Fig 6:**
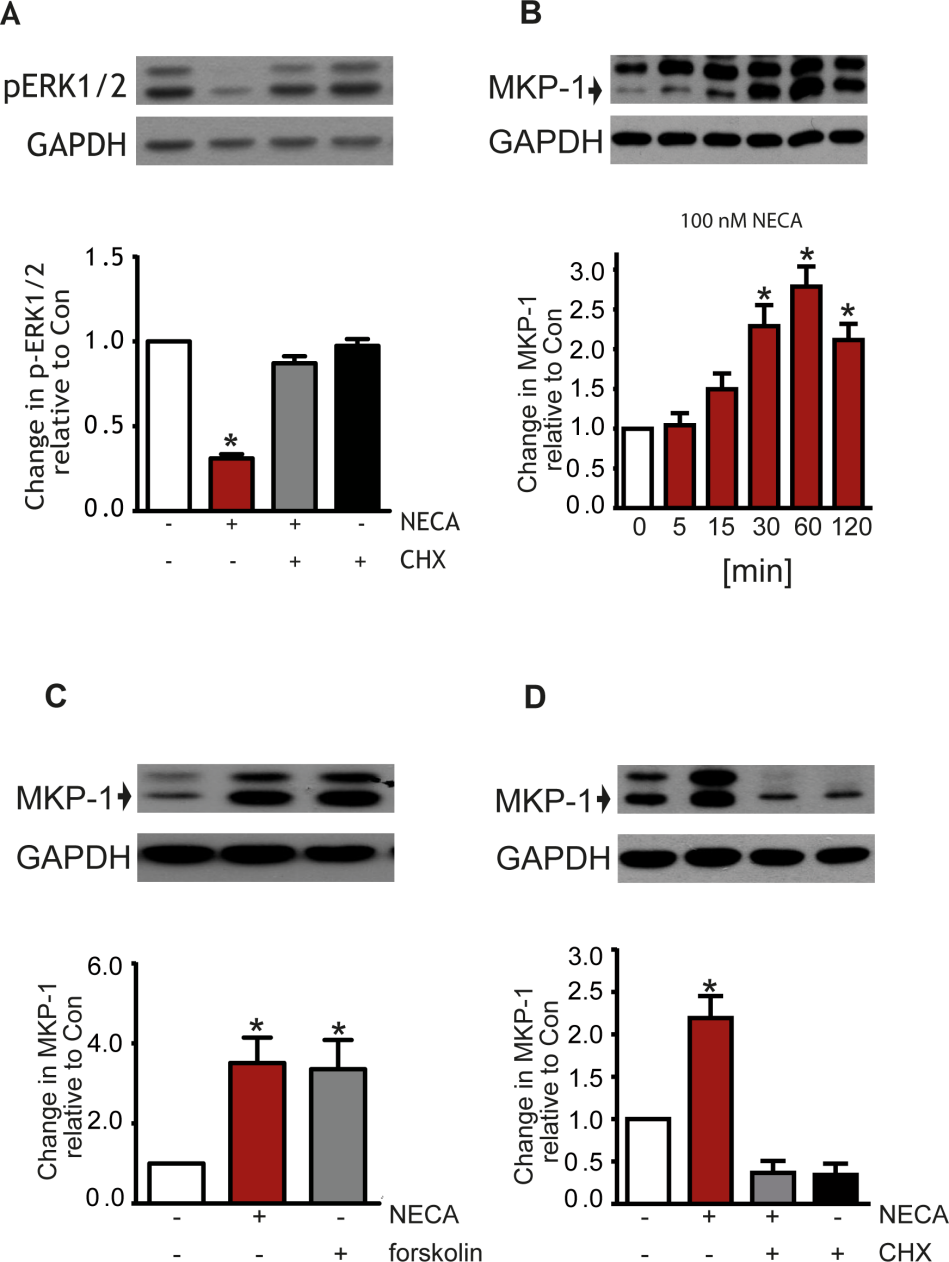
Effect of cycloheximide on ERK-1/2 phosphorylation and MKP-1 expression. The inhibition of ERK1/2 phosphorylation by NECA was blocked by 10 μg/ml of cycloheximide (CHX) suggesting that protein synthesis is mandatory for the NECA effect (A). Stimulation of A_2B_ receptors in MDA-MB-231 with NECA cells causes and increase in MKP-1 expression (B), treatment with 1 μM forskolin shows the same effect (C). The increase caused by NECA is blocked by cycloheximide (D). Western blots show a representative experiment, the columns represent mean values from n = 7 (A), 4 (B), 7 (C), and 6 (D) independent experiments (* p < 0.001, significantly different from control).

Inhibition of protein synthesis with cycloheximide abolished the NECA effect on ERK1/2 phosphorylation (Fig 6A). We identified MKP-1, a MAPK phosphatase, as a candidate whose expression might be involved in reduction of ERK1/2 phosphorylation by A_2B_ receptor stimulation with NECA. Fig 6B shows the time-dependent increase in MKP-1 expression upon stimulation with NECA. This effect was blocked by the addition of cycloheximide (Fig 6C) confirming a role of MKP-1 in the regulation of the phosphorylation state of ERK1/2.

**Fig 7:**
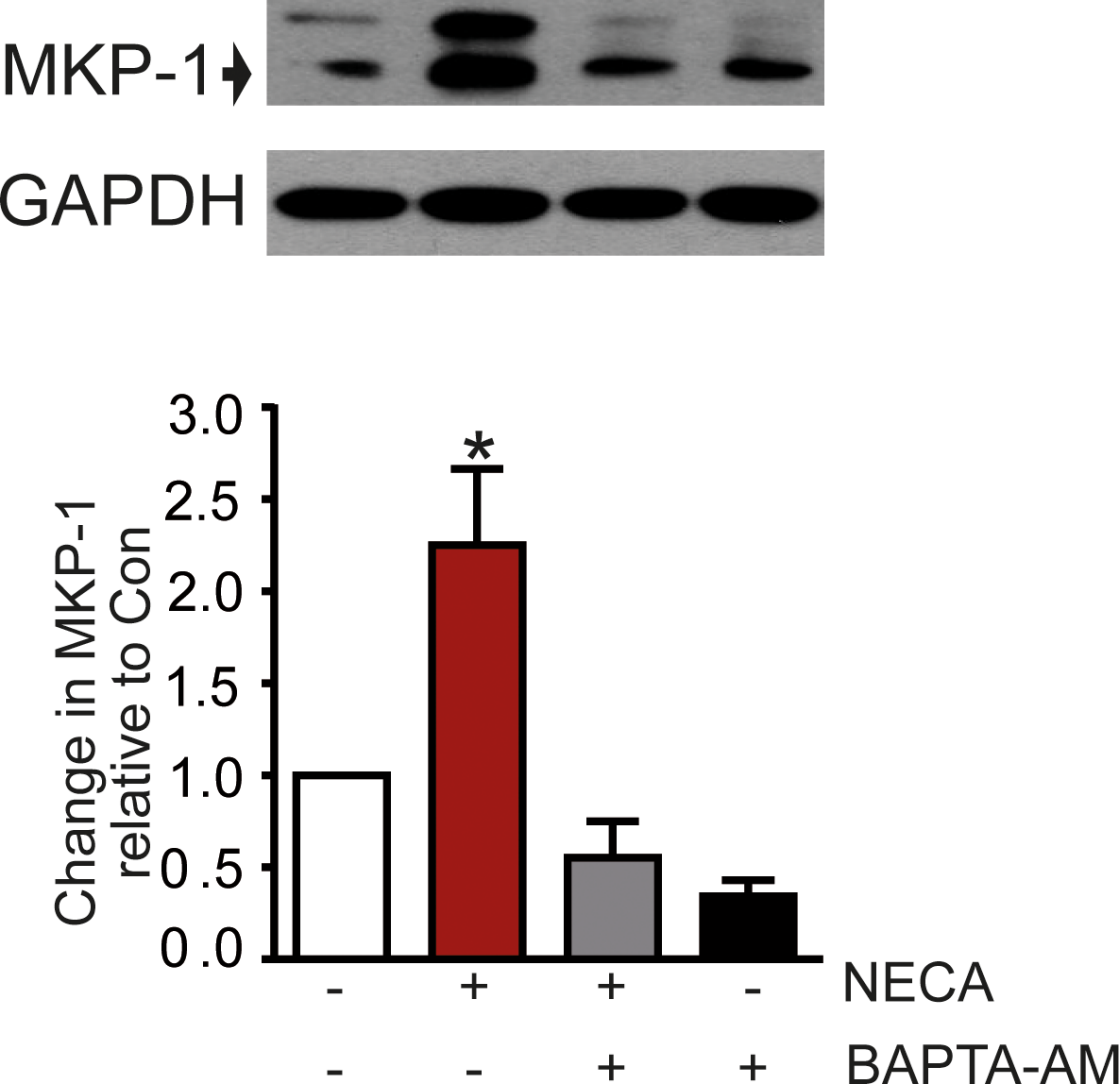
Ca^2+^ effect on MKP-1 expression. The NECA-induced increase of MKP-1 expression was blocked by the Ca^2+^-chelator BAPTA (addition of 30 μM BAPTA-AM). The Western blot shows a representative experiment, the columns represent mean values from n = 4 independent experiments (* p < 0.001, significantly different from control).

Regulation of MKP-1 activity was further established as a relevant mechanism contributing to the NECA-mediated reduction of ERK1/2 phosphorylation. The stimulation of adenylyl cyclase with forskolin resulted in an increased expression of MKP-1 similar to that seen after stimulation of the A_2B_ receptor with NECA (Fig 6D). The Ca^2+^-chelator BAPTA inhibited the NECA-effect on MKP-1 expression (Fig 7), suggesting that a Ca^2+^-signal may upregulate the level of MKP-1.

**Fig 8:**
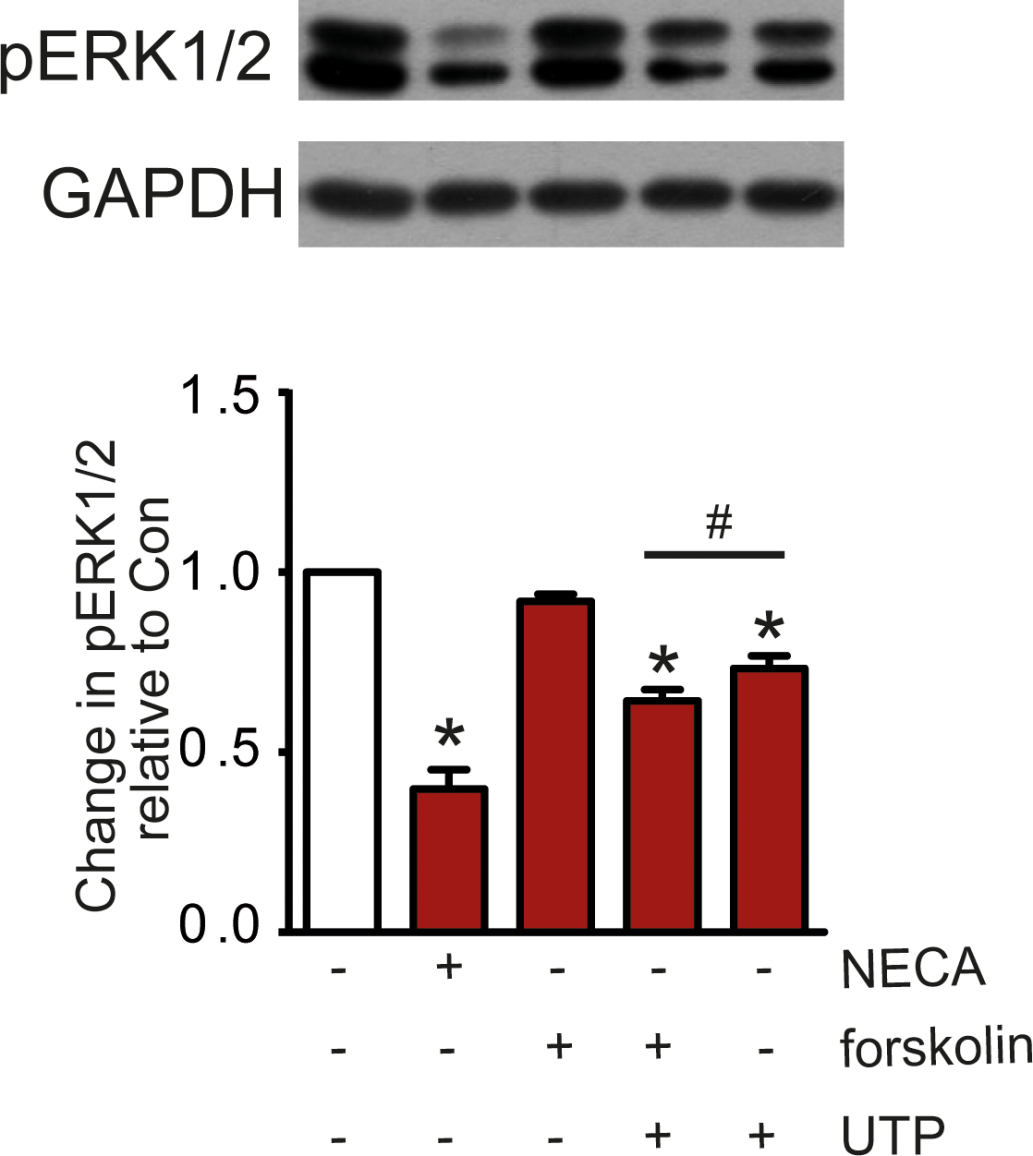
Reduction of ERK1/2 phosphorylation by UTP compared to NECA. The effect of 100 μM UTP was consistently lower than of 100 nM NECA. Addition of 30 nM forskolin to 100 μM UTP increased the UTP effect although forskolin at this concentration had no effect on its own. The Western blot shows a representative experiment, the columns represent mean values from from n = 7 independent experiments (* p < 0.001, significantly different from control; # p < 0.05, significantly different from each other).

Although Ca^2+^ is necessary for the NECA-mediated reduction of ERK1/2 phosphorylation it is not sufficient as is shown by the effect of UTP which elicited a Ca^2+^-signal via P2Y_2/4_ receptors (Fig 5B), but diminished ERK1/2 phosphorylation consistently to a lesser extent compared to NECA (Fig 5A and 5C). Given that the A_2B_ adenosine receptor triggers both an increase in cAMP and a Ca^2+^-signal it might be possible that these dual signals act in concert on the phosphorylation state of ERK1/2. Such a synergism was observed when combining UTP with a subthreshold concentration of forskolin (30 nM) with no effect on ERK1/2 phosphorylation on its own. In such an experiment forskolin increased the effect of UTP (Fig 8) supporting a role of synergism between signaling pathways regulating ERK1/2 phosphorylation.

**Fig 9:**
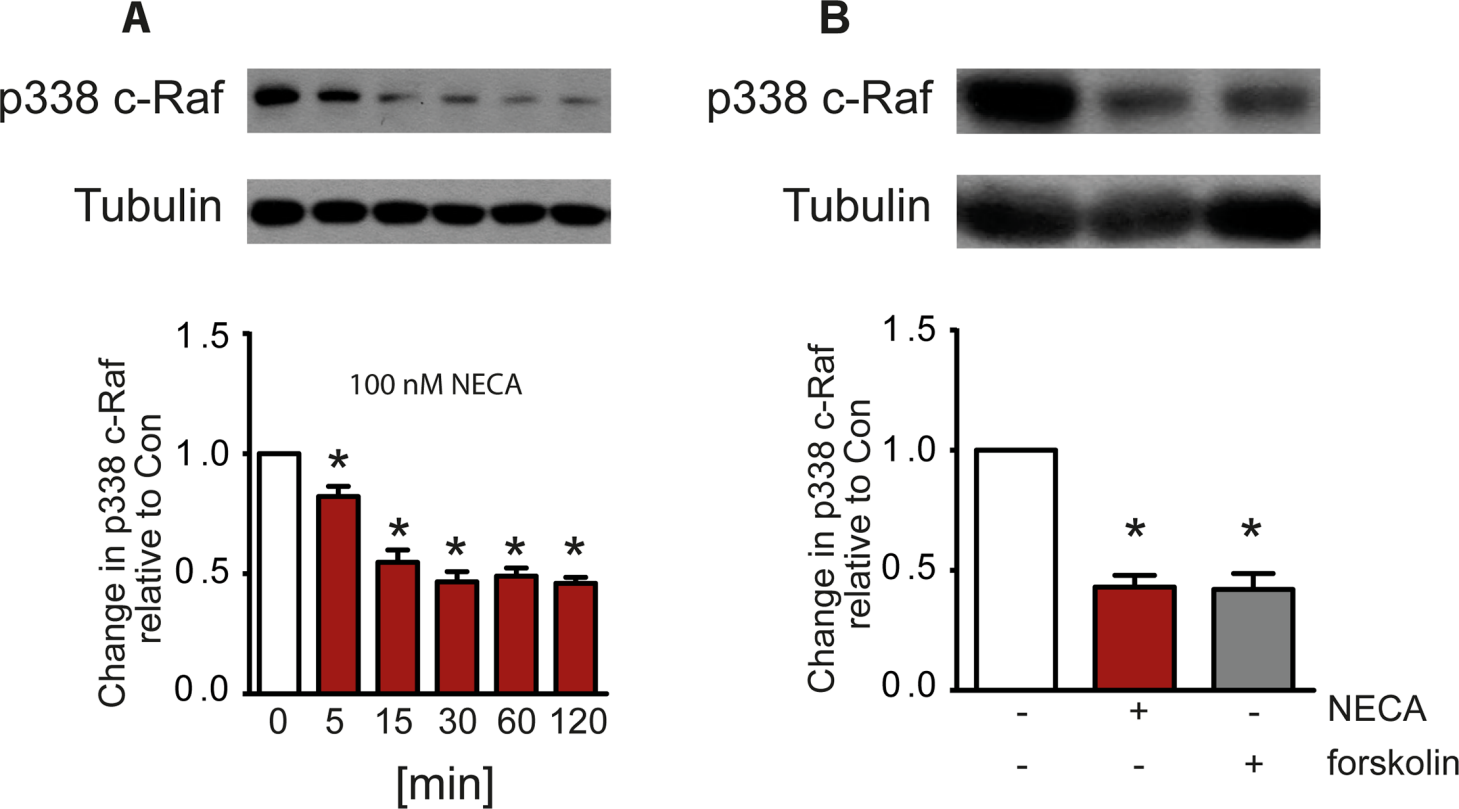
Phosphorylation of S338 of c-Raf is reduced by NECA and forskolin. A time-dependent inhibition of phosphorylation of c-Raf at S338 is observed by stimulation of the A_2B_ receptor with NECA (A). A similar effect was seen with forskolin (B). Western blots show a representative experiment, the columns represent mean values from n = 5 (A) and 10 (B) independent experiments (* p < 0.001, significantly different from control).

An additional contribution to a reduced phosphorylation of ERK1/2 by A_2B_AR stimulation was observed by another pathway leading to an A_2B_ receptor-dependent dephosphorylation of c-Raf. Stimulation of the A_2B_ receptor with NECA caused a time-dependent reduction of c-Raf phosphorylation on S338 which is a key activation site (Fig 9A). The same effect was observed after forskolin treatment suggesting a cAMP-dependent process (Fig 9B).

As a consequence of reduced ERK1/2 phosphorylation one would expect an effect on cell proliferation. S2 Fig confirms that stimulation of A_2B_ARs may reduce cell growth as shown by a reduced [^3^H]thymidine incorporation.

## Discussion

A plethora of data suggests that adenosine receptors play a role in the control of growth and proliferation of tumor cells [5, 6, 8]. In particular A_3_ adenosine receptors have been identified as important regulators of tumor progression [18–21]. Recently, we have shown that the estrogen receptor-negative cell line MDA-MB-231 expressed very high levels of A_2B_ adenosine receptors mediating both a stimulation of adenylyl cyclase and an activation of PLC with a consecutive Ca^2+^ signal [9]. Data from other cells show that the A_2B_ subtype may also stimulate the MAPK pathway [17] and thereby increase proliferation.

Attempts to identify a potential A_2B_-mediated stimulatory MAPK signal in MDA-MB-231 cells failed, probably due to high basal ERK1/2 phosphorylation in these cells. The surprising observation was that stimulation of A_2B_ adenosine receptors with the agonist NECA caused a reduction of the marked constitutive ERK1/2 phosphorylation. The effect occurred with the appropriate pharmacology that would be expected for an A_2B_AR-mediated response. Although reports exist of a receptor-mediated inhibition of MAPK signaling [22–24] the common observation is that G protein-coupled receptors mediate an activation of this pathway. The study presented here was initiated to understand the signaling cascade leading from A_2B_ activation to the reduction of ERK1/2 phosphorylation.

Our data reveal that multiple pathways are involved in this pathway as both cAMP as well as a Ca^2+^ signal is sufficient for a reduction of ERK1/2 phosphorylation to occur. The finding that protein synthesis is required for this inhibitory effect led to the hypothesis that a MAPK phosphatase (MKP) might be involved as the activity of these enzymes is subject to regulation by their expression level [25]. It was indeed observed that both signals triggered by stimulation of A_2B_AR with NECA (cAMP and Ca^2+^ signal) increased MKP-1 expression. It was previously established that intracellular Ca^2+^ is involved in the regulation of MKP-1 expression [26].

The reduction of ERK1/2 phosphorylation triggered by stimulation of an AR does not seem to be strictly dependent on A_2B_ARs. We could show that stimulation of the G_s_-coupled β adrenergic receptors with 100 nM isoproterenol caused in addition to the canonical activation of adenylyl cyclase a Ca^2+^ signal, in analogy to what was observed for A_2B_ARs (S3 Fig). Also in line with the effect observed for the A_2B_AR, stimulation of β adrenergic receptors resulted in a reduction of ERK1/2 phosphorylation (S3 Fig). It was recently reported that β_2_ receptors in HEK293 cells also trigger a cAMP-independent intracellular Ca^2+^ mobilization similar to the observation in MDA-MB-231 cell [27]. This result suggests that not just stimulation of A_2B_ARs may help to control tumor growth, the same effect might be exploitable in cancer cells expressing other G_s_-coupled receptors which in addition to an increase in cAMP mediate a concomitant Ca^2+^ signal.

It was striking that a Ca^2+^ signal as elicited by UTP via P2Y_2/4_ receptors caused a smaller reduction of ERK1/2 phosphorylation than stimulation of A_2B_ARs. This might be explained by the lack of a stimulatory effect of P2Y_2/4_ receptors on adenylyl cyclase as opposed to A_2B_AR signaling. Indeed, application of 30 nM forskolin, a concentration that had no effect on ERK1/2 phosphorylation of its own, along with UTP reduced ERK1/2 phosphorylation to the same degree as NECA, acting through A_2B_ARs. This result demonstrated that the cAMP signal and the Ca^2+^ signal synergistically reduced ERK1/2 phosphorylation. In addition, we could show that an A_2B_ receptor-mediated dephosphorylation of c-Raf at S338 also contributed directly to a reduction of ERK1/2 phosphorylation. A similar effect involving c-Raf was already observed in the past [28].

**Fig 10:**
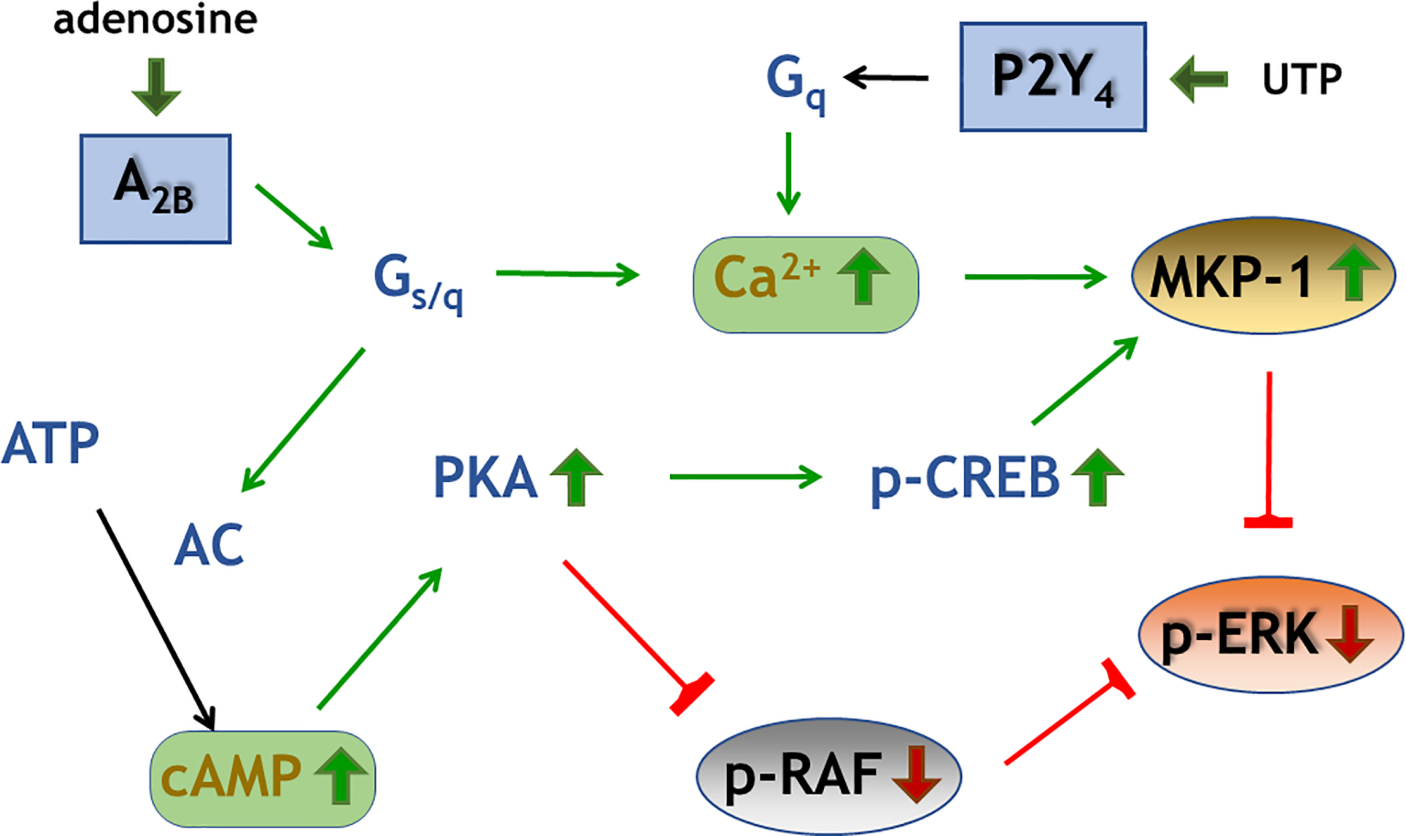
Overview of A_2B_ signaling pathways. The scheme summarizes signaling pathways contributing to a reduction of ERK1/2 phosphorylation through stimulation of A_2B_ adenosine receptors in MDA-MB-231 breast cancer cells. p-Raf denotes c-Raf phosphorylated at S338.

In summary, our data establish that activation of the A_2B_AR in MBA-MD-231 cells leads to an ERK1/2 dephosphorylation which is known to reduce cell proliferation as a result of inhibition of MAPK signaling. The A_2B_AR (and possibly other G_s_-coupled receptors) may be an attractive target for adjuvant treatment of tumors expressing this adenosine receptor subtype. It was found that MKP-1 played an important role for the reduction of ERK1/2 phosphorylation seen in the breast cancer cell line MBA-MD-231 after stimulation of A_2B_ARs. Several signaling pathways including an increase of cAMP or intracellular Ca^2+^ lead to an increased expression of MKP-1, thus, resulting in a reduction of ERK1/2 phosphorylation (Fig 10).

## Supporting information

S1 Fig GAPDH as loading control in Western blots. Unphosphorylated ERK1/2 could not successfully be used as a loading control as the intensity of the staining with both ERK1/2 and pERK1/2 antibodies prevented sufficient stripping of the labeled bands before labeling the overlapping second set of bands. It is shown that GAPDH and ERK1/2 staining (same gels) result in identical documentation of equal loading of the gels. pERK1/2 labeling is shown with the same samples on a separate gel for comparison. The columns show the means of n = 5 experiments with SEM; * p < 0.001.
(PDF)

S2 Fig NECA inhibits proliferation of MDA-MB-231 cells. [^3^H]Thymidine incorporation was determined as described earlier (S1 Text). It is shown that cell proliferation is inhibited to about 80% after 12 and 24h of incubation with NECA. Data show mean values with SEM of n = 8 experiments performed in triplicates. The NECA values are significantly different from controls with p = 0.0011 and 0.0004 for the 12 and 24h time points, respectively (paired Student’s t-test).
(PDF)

S3 Fig Isoproterenol triggers a Ca^2+^ signal and reduces ERK1/2 phosphorylation. (PDF)

S1 Text Reference. (DOCX)
