# Supplementary figures and images for: The A2B adenosine receptor in MDA-MB-231 breast cancer cells diminishes ERK1/2 phosphorylation by activation of MAPK-phosphatase-1
Source: PLoS One. 2018 Aug 29;13(8):e0202914. doi: 10.1371/journal.pone.0202914 (PMC6114864; doi:10.1371/journal.pone.0202914)

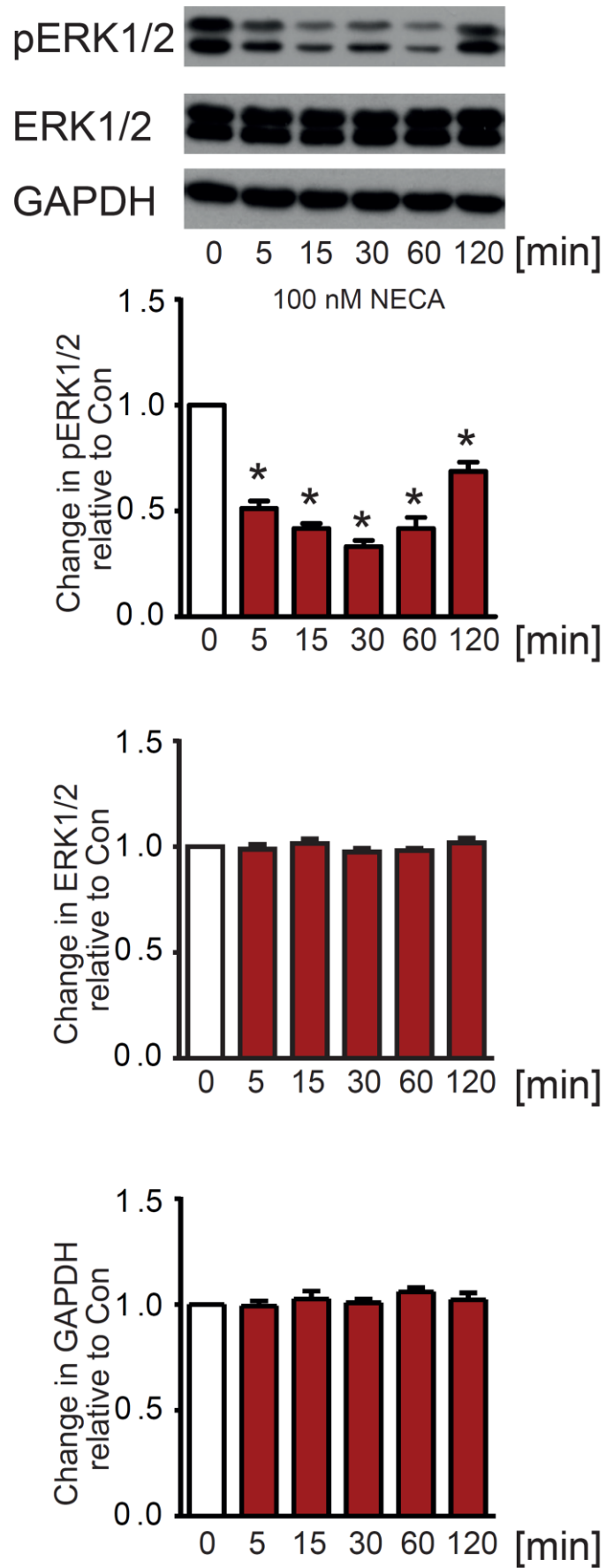

S1 Fig

Supplement: S1 Fig — Unphosphorylated ERK1/2 could not successfully be used as a loading control as the intensity of the staining with both ERK1/2 and pERK1/2 antibodies prevented sufficient stripping of the labeled bands before labeling the overlapping second set of bands. It is shown that GAPDH and ERK1/2 staining (same gels) result in identical documentation of equal loading of the gels. pERK1/2 labeling is shown with the same samples on a separate gel for comparison. The columns show the means of n = 5 experiments with SEM; * p < 0.001. (PDF) [file pone.0202914.s001.pdf]

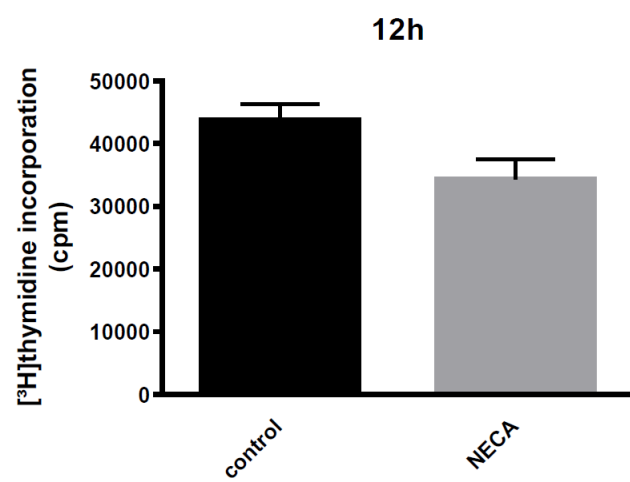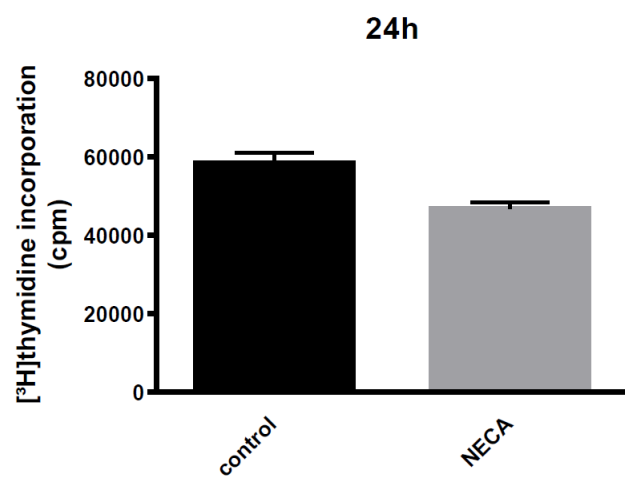

S2 Fig

Supplement: S2 Fig — [3H]Thymidine incorporation was determined as described earlier (S1 Text). It is shown that cell proliferation is inhibited to about 80% after 12 and 24h of incubation with NECA. Data show mean values with SEM of n = 8 experiments performed in triplicates. The NECA values are significantly different from controls with p = 0.0011 and 0.0004 for the 12 and 24h time points, respectively (paired Student’s t-test). (PDF) [file pone.0202914.s002.pdf]

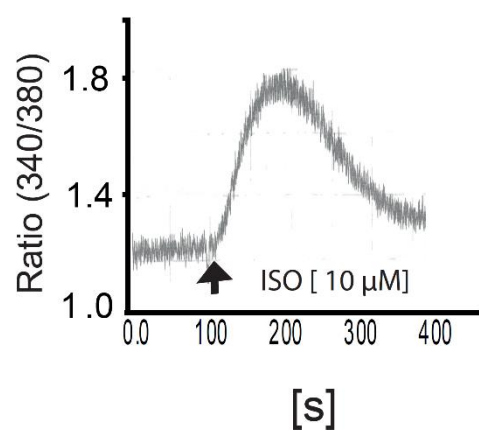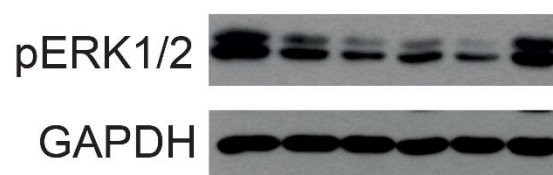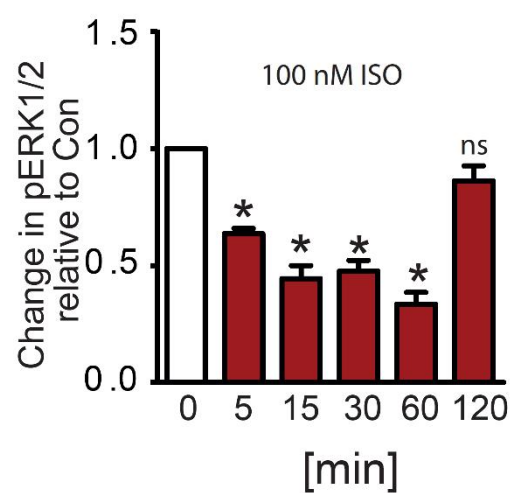

**S3 Fig**

Supplement: S3 Fig — (PDF) [file pone.0202914.s003.pdf]
