# Supplementary material for: The A2B adenosine receptor in MDA-MB-231 breast cancer cells diminishes ERK1/2 phosphorylation by activation of MAPK-phosphatase-1
Source: PLoS One. 2018 Aug 29;13(8):e0202914. doi: 10.1371/journal.pone.0202914 (PMC6114864; doi:10.1371/journal.pone.0202914)
Supplement: S1 Text — (DOCX) [file pone.0202914.s004.docx]

Reference:

Schmid E, Neef S, Berlin C, Tomasovic A, Kahlert K, Nordbeck P, et al. Cardiac RKIP induces a beneficial β-adrenoceptor-dependent positive inotropy. Nat Med. 2015,21: 1298-1306.
